# Quality of life after transoral CO_2_ laser posterior cordotomy with or without partial arytenoidectomy for bilateral adductor vocal cord paralysis

**DOI:** 10.1007/s00405-021-06971-7

**Published:** 2021-07-18

**Authors:** Marta Filauro, Alberto Vallin, Elisa Marcenaro, Francesco Missale, Marco Fragale, Francesco Mora, Valeria Marrosu, Claudio Sampieri, Filippo Carta, Roberto Puxeddu, Giorgio Peretti

**Affiliations:** 1 Unit of Otorhinolaryngology-Head and Neck Surgery, IRCCS Ospedale Policlinico San Martino, Largo Rosanna Benzi, 10, 16132 Genova, Italy; 2 Department of Surgical Sciences and Integrated Diagnostics (DISC), University of Genova, Genova, Italy; 3 Department of Experimental Medicine (DIMES), University of Genova, Genova, Italy; 4 Department of Molecular and Translational Medicine, University of Brescia, 25121 Brescia, Italy; 5 Department of Otorhinolaryngology-Head and Neck Surgery, University of Cagliari, 09124 Cagliari, Italy

**Keywords:** Vocal fold paralysis, Glottis, Larynx, Laser, Cordotomy

## Abstract

**Objectives:**

Bilateral adductor vocal cord paralysis (BAVCP) is a rare and challenging condition whose main consequence is reduction of airway patency at the glottic level, often causing respiratory distress, while vocal function tends to remain almost normal. We investigated the effect of transoral glottal widening on quality of life and decannulation rates in patients affected by BAVCP.

**Methods:**

We retrospectively evaluated patients affected by BAVCP and treated by transoral CO_2_ posterior cordotomy with or without medial partial arytenoidectomy (PC ± MPA) at two referral centers. The primary outcome was change in quality of life, evaluated pre- and post-operatively by the ADVS, VHI-30, and EAT-10 questionnaires. Secondary outcomes were the need for retreatments and, for patients with tracheotomy, the time to decannulation.

**Results:**

Thirty-three patients met selection criteria. The etiology was post-surgical in 27 cases (81.8%), idiopathic in 4 (12.1%), a trauma-related in 1 (6.0%), and to other causes in 1 (3.0%). In 22 cases (66.7%), PC was combined with MPA. A significant improvement in responses for the ADVS (*p* < .0001) and EAT-10 (*p* < .0001) was observed, whereas the VHI-30 score did not change significantly post-operatively. All nine patients with a tracheostomy were successfully decannulated within 18 months after the surgical procedure.

**Conclusions:**

For patients affected by BAVCP, PC ± MPA by transoral CO_2_ laser microsurgery is a safe, customizable and minimally invasive treatment that can guarantee an affordable balance between quality of life in terms of phonation and swallowing and acceptable airway patency.

## Introduction

Bilateral adductor vocal cord paralysis (BAVCP) is an uncommon but challenging problem for the head and neck surgeon. BAVCP is the most common neurological disorder of the larynx and may result from vagal or recurrent laryngeal nerve damage or from a central lesion [1]. The most frequent etiology of BAVCP is iatrogenic trauma (44%), and the majority are related to thyroid surgery; other reported causes are malignancies (17%), endotracheal intubation (15%), neurologic diseases (12%), and idiopathic (12%) [2]. The clinical profile of the patient affected by BAVCP is quite variable: at onset, a patient may complain of a breathy voice without respiratory fatigue; progressively, vocal performance may even improve, while respiratory symptoms worsen. Patients often show severe dyspnea or stridor with no obvious voice disorders [3]. However, to date, in the medical community general agreement on the surgical treatment that will grant patients the best quality of life is still lacking.

The ideal surgical option should ensure a better quality of life by relieving airway obstruction and preserving laryngeal functions such as phonation and deglutition. Until the 1920s, tracheotomy was the only treatment routinely applied, while at present, its application is mainly limited to emergency situations to provide immediate respiratory relief. Nowadays, a wide spectrum of surgical procedures by transoral or open-neck approaches are available [4–10]. Among these, transoral CO_2_ laser posterior cordotomy is one of the most popular surgical techniques, due to the low rates of complications, absence of external scar, and short surgical and hospitalization times [11, 12].

The purpose of this multicenter retrospective study is to evaluate long-term outcomes of transoral microsurgical treatments of glottic stenosis due to BAVCP using both objective methods and symptom-related quality-of-life questionnaires.

## Materials and methods

### Patient cohort

A retrospective observational study was carried out enrolling patients affected by BAVCP and treated by transoral CO_2_ PC ± MPA from September 2013 to April 2019 at two Italian referral centers: the Otolaryngology - Head and Neck Surgery Department of the IRCCS Ospedale Policlinico San Martino in Genova, and the Otolaryngology – Head and Neck Surgery Department of the University Hospital of Cagliari. As inclusion criteria, patients affected by BAVCP and treated by posterior cordotomy ± medial partial arytenoidectomy by transoral CO_2_ laser microsurgery were selected (*n *= 43). Patients with missing data on pre- and post-operative videolaryngostroboscopy and quality-of-life assessment were excluded from the analysis (*n *= 10).

### Clinical evaluation

Diagnosis of BAVCP was made using an angled rigid telescope, performed in the office with a 70° rigid endoscope and a Kay Digital Strobe 9200 (Kay Elemetrics Co., Pine Brook, NJ, USA) or a flexible videoendoscope coupled with an OLYMPUS CLL-S1 Strobe LED light source (Olympus, Tokyo, Japan). Full laryngological and neurological examination was performed before surgery and the minimum interval between diagnosis of BAVCP and surgical planning was at least 6 months.

### Surgical technique

**Fig. 1:**
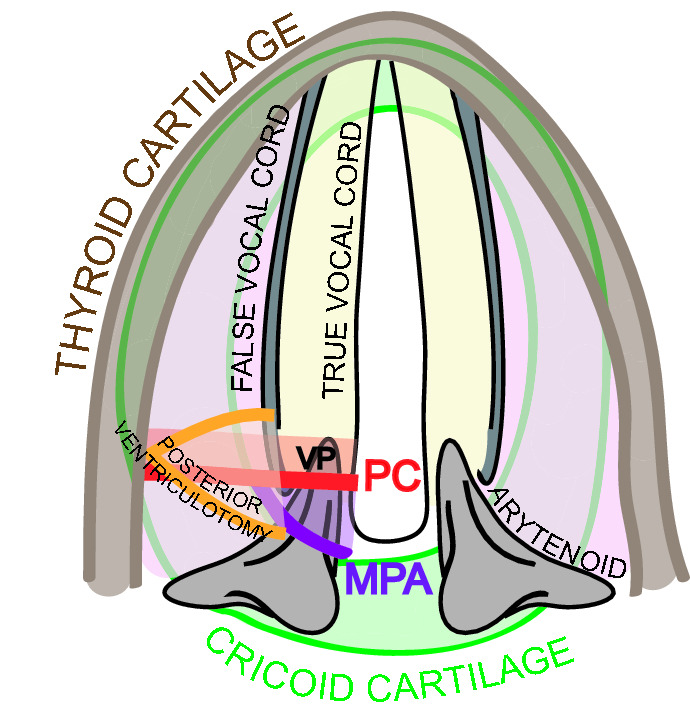
Draw that illustrates the surgical techniques adopted. *VP* vocal process, *PC* posterior cordotomy, *MPA* medial partial arytenopidectomy

**Fig. 2:**
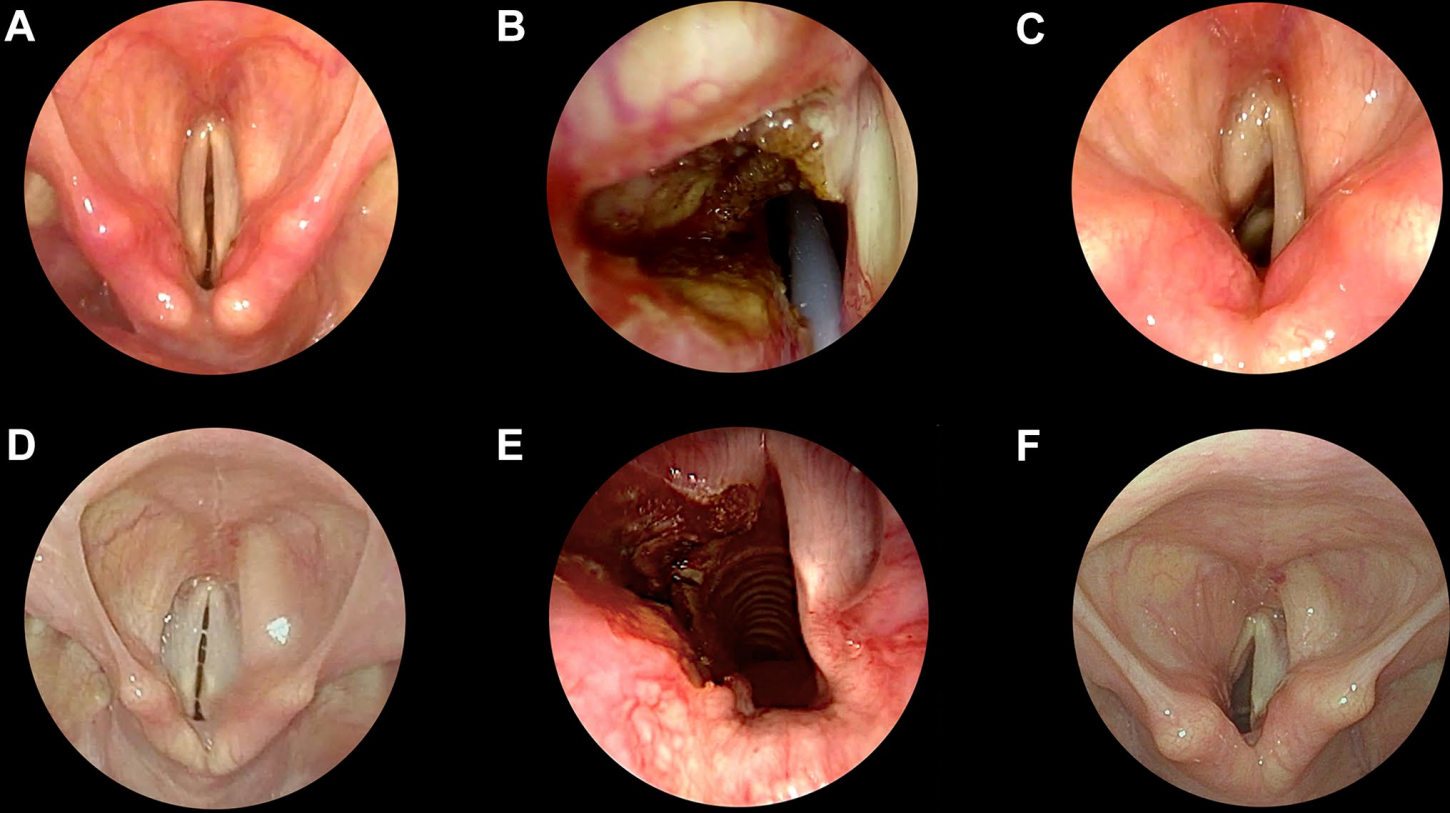
Endoscopic pre-treatment (**A**, **D**), intraoperative (**B**, **E**) or post-treatment (**C**, **F**) pictures of two clinical cases managed with transoral CO_2_ laser posterior cordotomy alone (**A–C**) or with medial partial arytenoidectomy (**D–F**)

All patients were treated by CO_2_ transoral laser microsurgery (TLM) by two senior surgeons (G.P and R.P). TLM procedures were performed under general anesthesia with a Lumenis Encore Ultrapulse (Tel Aviv, Israel) coupled with a digital Acublade micromanipulator set at 2–10 W of power, delivered in an ultrapulse modality and continuous mode. Patients without tracheostomy were intubated with an orotracheal tube 5.0–6.0 mm in internal diameter (Shiley^™^ Laser Oral Endotracheal Tubes, Medtronic Xomed, Jacksonville, FL, USA) or with infraglottic high frequency jet ventilation (Monsoon III, Acutronic, Switzerland and TwinStream, Carl Reiner GMBH, Austria) using a transglottic double lumen laser catheter made of incombustible tetrafluoroethylene, with an outer diameter of 4 mm [13]. Tracheostomized patients were intubated through the tracheostoma. Wet sponges were placed under the glottic plane to protect the cuff and subglottic structures from thermal lesions, except in tubeless procedures. Laryngeal exposure was obtained in the Boice–Jackson position using a wide gamma of different laryngoscopes, depending on patient’s anatomical variables conditioning the exposure, which was preoperatively evaluated by the Laryngoscore [14]. The surgical procedure began with a unilateral partial ventriculectomy to remove the posterior third of the false vocal fold to achieve adequate exposure of the posterior third of the true vocal cord and the floor of the ventricle. Afterwards, a CO_2_ laser incision of the mucosa of the true vocal fold including the vocal process was performed. The CO_2_ laser cut was then extended through both vocalis and muscularis parts of the thyro-arytenoid muscle, up to the inner perichondrium of thyroid lamina, laterally. Caudally, the section was extended up to the superior margin of cricoid. Afterwards, coarctation and adjunctive retraction of the posterior part of the thyro-arytenoid muscle was done by laser photocoagulation, aimed at improving the post-treatment abduction of the vocal cord after the healing process (PC) (Figs. 1, 2A–C). If deemed necessary, during the patient’s counseling, a medial partial arytenoidectomy (MPA) was simultaneously combined to achieve a wider posterior respiratory space (Figs. 1, 2D–F). MPA was performed, sparing the muscular process and the postero-lateral part of the body of the arytenoid, keeping intact the hypopharyngo-laryngeal posterior wall to prevent aspiration during the swallow.

### Quality-of-life assessment

Quality of life was considered the primary outcome and measured pre- and post-treatment (at least 6 months after the surgical procedure) by the Voice Handicap Index (VHI), eating assessment tool (EAT-10), and Airway-Dysphonia-Voice-Swallowing (ADVS) staging system.

The VHI-30 is a 30-item questionnaire on the patient’s voice-related issues, equally partitioned in three domains: emotional, functional, and physical. Patients subjectively assessed each query using a 5-point scale ranging from 0 (never) to 4 (always) [15, 16].

Swallowing function was evaluated by the EAT-10, a ten-item self-assessment questionnaire validated as dysphagia symptom-specific outcome measure that evaluates symptom severity, quality of life, and treatment efficacy [17, 18]. Patients were asked to rate several swallowing issues on a Likert scale from 0 to 4 where 0 = no problem and 4 = severe problem, and the maximum severity score is 40. Normative data suggest that an overall EAT-10 score of 3 or more is abnormal.

ADVS assessment was carried out to evaluate a disease-specific patient-reported outcome measure [19, 20]. It is a proven system that portrays the functional outcome of adult and children laryngotracheal stenosis. It examines four domains [airway (A), dyspnea (D), voice (V) and swallowing (S)] established by the physician and patient. All domains have a 1–5 scale, from least to most serious.

### Airway evaluation

At least 6 months after surgical treatment objective ventilatory function was evaluated on the basis of the flow-volume loop obtained by a spirometer. Pulmonary function test (PFT) was performed on all patients measuring the post-operative peak expiratory flow (PEF), a well-established objective parameter to evaluate the extent of residual airway obstruction [21–23]. The decannulation rate was a secondary outcome, measurable for the cohort of patients with a tracheostomy at the time of the surgical procedure (*n *= 12, 30%).

### Statistical analysis

Clinical data were summarized with absolute and relative frequencies for categorical data and summary statistics for numeric variables as mean, standard deviation (SD), median, and range. Paired comparisons of questionnaires results were performed by Wilcoxon Signed Rank test, as appropriate. Survival analysis for time to decannulation or time to re-treatment was performed with the Kaplan–Meier and log-rank test. The effect of the timing variable (pre-treatment, post-treatment) for the quality-of-life results was investigated with two-way repeated ordinal regressions and ANOVA type II Sums of Squares analysis [24], testing the interaction with possible confounders: etiology and choice of a simultaneous MPA. In all analyses, a two-tailed p value < 0.05 was considered significant. GraphPad Prism (San Diego, CA, USA) and R (version 3.6.2) were used for statistical analysis.

## Results

### Clinical features

Thirty-three patients, 9 males (27.3%) and 24 females (72.7%) met inclusion criteria and were available for the analysis; 10 patients were excluded for missing data on preoperative or postoperative evaluations. The average age was 56.2 years (range 11–77). The etiology was iatrogenic post-surgery in 27 cases (81.8%), idiopathic in 4 (12.1%), sequelae of laryngeal trauma in 1 (3.0%), and associated with skull base trauma and post-surgery in 1 case (3.0%). Nine patients (27.3%) underwent tracheotomy before or during the posterior cordotomy procedure if deemed necessary. In 22 patients (66.7%), a simultaneous MPA was performed. Six patients required revision surgery due to recurrent dyspnea: in five cases, a revision posterior cordotomy on the same side was performed, and in one case, a contralateral posterior cordotomy was added to ensure adequate glottal widening. Summary statistics of demographic and clinical features are presented in Table 1.

**Table 1:** Clinical features of the cohort of patients

	Overall (*n *= 33)
Hospital	
Center 1 (Genoa)	19 (57.6%)
Center 2 (Cagliari)	14 (42.4%)
Age	
Mean (SD)	56.2 (15.1)
Median [min, max]	59.0 [11.0, 77.0]
Gender	
F	24 (72.7%)
M	9 (27.3%)
Etiology	
Post-surgery	27 (81.8%)
Idiopathic	4 (12.1%)
Trauma	1 (3.0%)
Post-surgery and Trauma	1 (3.0%)
Medial partial arytenoidectomy	
No	11 (33.3%)
Yes	22 (66.7%)
Jet ventilation	
No	26 (78.8%)
Yes	7 (21.2%)
Tracheostomy	
No	24 (72.7%)
Yes	9 (27.3%)
Retreatments	
No	27 (81.8%)
Yes	6 (18.2%)
Decannulation	
Yes	9 (27.3%)
No	0 (0%)
No tracheostomy	24 (72.7%)

### Questionnaire results

**Fig. 3:**
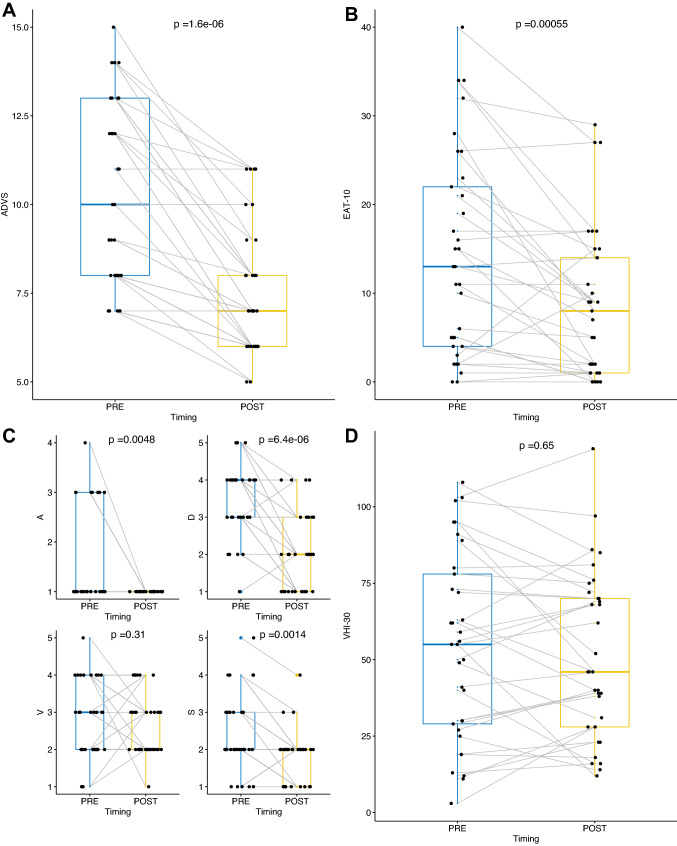
Box plots of ADVS (**A, C**), EAT-10 (**B**), andVHI-30 (**D**) questionnaire results; *p* values estimated by Wilcoxon test

The quality of life measured with the ADVS and EAT-10 questionnaires significantly improved, with mean ADVS score variation (ADVS_POST_ − ADVS_PRE_) of − 2.85 (CI_95%_ − 3.57, − 2.12; *p* < 0.0001) and EAT-10 score variation (EAT-10_POST_ − EAT-10_PRE_) of − 5.42 (CI_95%_ − 8.30,  − 2.55; *p* = 0.0005). Furthermore, significant improvement of all of the items of the ADVS questionnaires was observed, as extensively reported in Table 2 and Fig. 3A–C. No significant changes in the VHI-30 score were observed with a mean variation (VHI-30_POST_ − VHI-30_PRE_) of − 3.12 (CI_95%_ − 9.80, + 3.56; p = 0.65), as reported in Table 2 and Fig. 3D.

**Table 2:** Summary statistics of preoperative (PRE), postoperative (POST) questionnaire results and the mean actual difference of paired evaluations (POST–PRE); *p* values by Wilcoxon test

	PRE	POST	Mean difference _POST–PRE_ (CI_95%_)	*p*
A				
Mean (SD)	1.58 (0.969)	1.00 (0)	− 0.58 (− 0.92, − 0.23)	0.005
Median [Min, Max]	1.00 [1.00, 4.00]	1.00 [1.00, 1.00]		
D				
Mean (SD)	3.55 (1.00)	2.09 (1.01)	− 1.45 (− 1.84, − 1.07)	< 0.0001
Median [min, max]	4.00 [1.00, 5.00]	2.00 [1.00, 4.00]		
V				
Mean (SD)	2.85 (1.00)	2.64 (0.822)	− 0.21 (− 0.61, +0.18)	0.31
Median [min, max]	3.00 [1.00, 5.00]	2.00 [1.00, 4.00]		
S				
Mean (SD)	2.39 (0.998)	1.82 (0.683)	− 0.58 (− 0.88, − 0.27)	0.001
Median [min, max]	2.00 [1.00, 5.00]	2.00 [1.00, 4.00]		
ADVS				
Mean (SD)	10.4 (2.57)	7.52 (1.80)	− 2.85 (− 3.57, − 2.12)	< 0.0001
Median [min, max]	10.0 [7.00, 15.0]	7.00 [5.00, 11.0]		
EAT-10				
Mean (SD)	14.1 (11.4)	8.67 (8.40)	− 5.42 (− 8.30, − 2.55)	0.0005
Median [min, max]	13.0 [0, 40.0]	8.00 [0, 29.0]		
VHI-30				
Mean (SD)	54.4 (30.5)	51.3 (27.2)	− 3.12 (− 9.80, +3.56)	0.65
Median [min, max]	55.0 [3.00, 108]	46.0 [12.0, 119]		
PEF (%predicted)				
Mean (SD)	–	53.7 (21.5)	–	–
Median [min, max]	–	52.5 [12.2, 96.0]		
Missing	–	5 (15.2%)

### Quality-of-life predictors

**Fig. 4:**
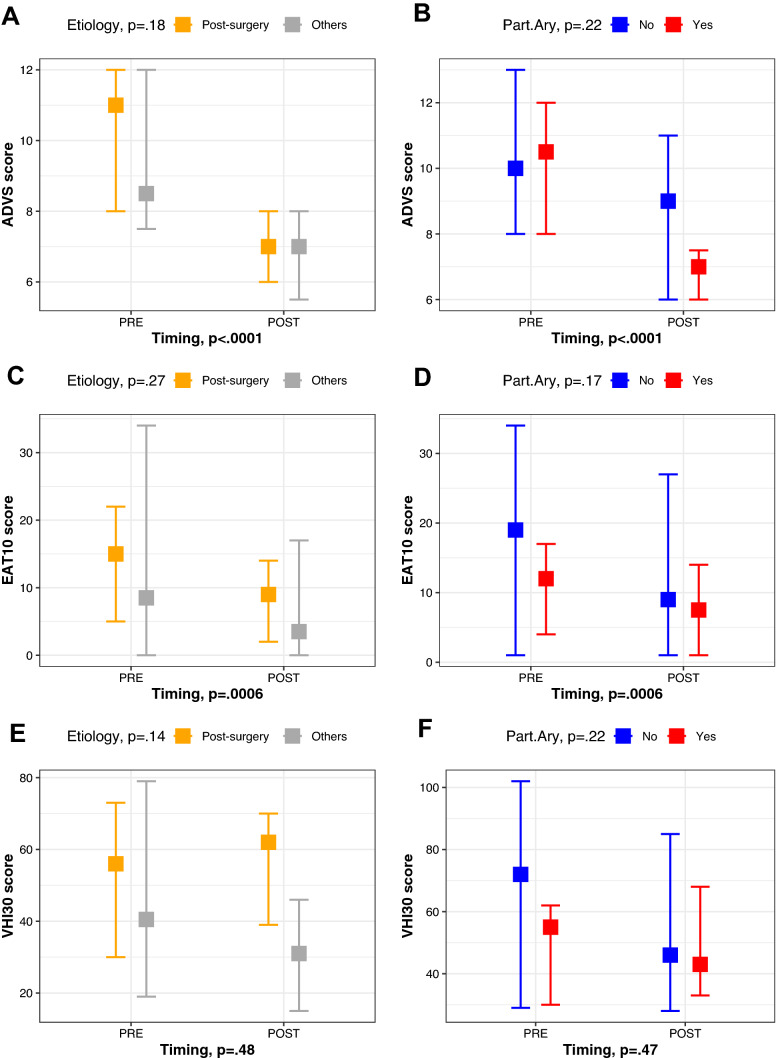
Plots showing pre-treatment (PRE) and post-treatment (POST) median values with 95% CI of ADVS scores (**A**, **B**), EAT-10 scores (**C**, **D**), and VHI-30 scores (**E**, **F**) in different subgroups of patients according to etiology and synchronous partial arytenoidectomy. The main effect of each variable was tested by ordinal regression models, weighed by the timing effect (PRE vs POST); *p* values estimated by ANOVA type II Sums of Squares analysis

Two-way ordinal regression models were built testing the effect of different etiology (post-surgical vs others) and execution of a synchronous partial arytenoidectomy on the overall ADVS, VHI-10, or EAT-10 post-treatment effect (Timing), as shown in Fig. 4. Significant post-treatment improvement of ADVS (*p* < 0.0001) and EAT-10 (*p* = 0.0006) scores was confirmed, weighted for either different etiology or execution of a synchronous MPA. Performing a MPA was not associated with different scores, or a different etiology, weighted for the timing effect (*p* > 0.05), as shown in Fig. 4E.

### Retreatments

**Fig. 5:**
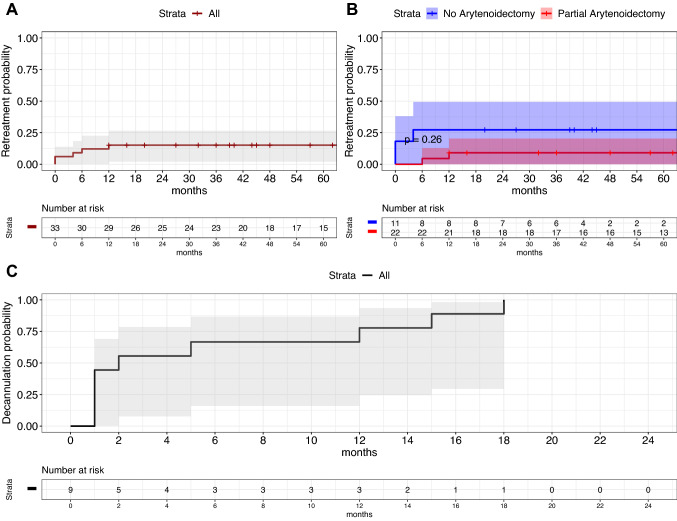
Kaplan–Maier curves showing the probability of retreatments for the whole cohort (**A**) or stratified by synchronous partial arytenoidectomy (**B**) and decannulation probability for the subgroup of patients with a tracheotomy at the time of the endoscopic procedure (**C**); *p* value estimated by log-rank test

The estimated risk of retreatments at 1, 2, and 5 year was 15.0% (CI_95%_ 2.0–27.0%), 15.0% (CI_95%_ 2.0–27.0%), and 15.0% (CI_95%_ 2.0–27.0%), respectively, as shown in Fig. 5A. The execution of a synchronous partial arytenoidectomy was not associated with a significantly lower rate of retreatments (*p* = 0.26), although a trend of lower risk was observed with estimated risks at 2 years of 9.1% (CI_95%_ 0.0–20.0%) compared to 27.0% (CI_95%_ 0.0–49.0%) (Fig. 5B).

### Decannulation and airway evaluation

All nine patients with a tracheostomy at the time of the endoscopic widening procedure were successfully decannulated within 18 months with a probability of decannulation at 6 and 12 months of 67% (CI_95%_ 16–87%) and 78% (CI_95%_ 25–93%), as shown in Fig. 5C. In our cohort, the postoperative mean PEF% value was 53.7% ± 21.5, with a median of 52.5%.

## Discussion

BAVCP is a potentially life-threatening condition needing surgical treatment in most cases. Tracheotomy represents an emergency front-line approach for BAVCP to immediately restore the patency of the airway. Undoubtedly, due to its invasiveness, complications, stigma, and quality-of-life issues, it cannot be considered as the most appropriate therapeutic solution. In alternative, several surgical techniques for the management of BAVCP have been reported in the literature. In 1922, Chevalier Jackson was the first surgeon to perform open ventriculocordectomy with the intent of preserving the voice. Even if this procedure gave an optimal airway, voice quality was poor [4]. Arytenoidectomy by a cervical approach, outlined by Woodman et al. also obtained ample accreditation [5].

Transoral arytenoidectomy was later described by Thornell et al., in 1948 [6]. For the first time, Ossoff et al. introduced the use of laser during transoral arytenoidectomy [7]. Transoral external vocal fold laterofixation with sutures did not gain wide acceptance due to technical difficulties, customized instrumentation, and success rates that were not reproducible [8]. In 1991, Kashima et al. [25] described the endoscopic transvers posterior cordotomy technique and in 1993 Crumley [26] proposed endoscopic laser medial arytenoidectomy as an alternative to previous techniques to achieve glottic enlargement without affecting the quality of voice. In addition, subtotal arytenoidectomy combined with posterior true and false cordotomy has also been proposed [9]. Lastly, bilateral laryngeal selective reinnervation represents a new and promising surgical technique for BAVCP, which could possibly be an alternative, in the near future, to static and irreversible surgical procedures [10]. As described by Marina et al.[10], this technique aims at giving a newly motor innervation trough a “Y”-shaped nerve graft between the first phrenic root and both posterior crico-arythenoid muscles to ensure abduction of vocal cords.

To date, for the treatment of non-reversible BAVCP, transoral laser-assisted PC is still more frequently used and preferred to external techniques for its adjustability to the patient’s needs, lower complication rates, absence of external scar, and shorter surgical and hospitalization times. Our technique described herein, differs from the Dennis and Kashima one [25], because we routinely carry out the resection of the posterior third of false vocal fold including the vocal process of the arytenoid and removing the posterior third of the vocal cord, to gain wider respiratory space. In addition, we vaporize the posterior part of thyroarytenoid muscle, sparing the overlining ligament and mucosa to obtain a late lateral scarring retraction of the entire vocal fold, without shortening the length of its medial surface. Moreover, if during pre-operative counseling breathing function emerges to be essential compared to the speaking one, the surgical procedure can be tailored to the needs of each patient by combining a MPA with the previous PC to ensure a potential wider glottic airway space.

As previously reported, this type of surgical procedure does not require prophylactic tracheotomy, thereby minimizing post-operative morbidity and hospitalization time and improving vocal outcomes [27, 28]. Our data, in line with that reported in the literature [29, 30], show that CO_2_ laser PC ± MPA does not negatively affect swallowing function, although it does cause wide unilateral posterior glottic incompetence, as this is the aim of the surgical procedure itself. A number of different scales (i.e. PAS, FOSS, FOIS) have been used to assess potential dysphagia worsening due to glottic widening [31–33]. By the EAT-10 (*p* < 0.0001) and S item of the ADVS (*p *= 0.001) questionnaires, we observed significant improvement of subjective dysphagia; possible explanations of this positive outcome could lie in the successful decannulation of all patients who were tracheostomized, allowing restoration of the physiologic coordination between swallowing maneuvers and breathing [34][34]. In this regard, Yilmaz et al. [30], in agreement with our results, reported that partial arytenoidectomy, in patients affected by BAVCP, did not negatively interfere with swallowing functions. Furthermore, is evident that laryngeal sphincteric function is the result of a more complex physiologic mechanism, wherein glottic closure by TA muscles contraction is only a part of the entire process. Consequently, if the approximation of false vocal folds and arytenoids to epiglottic petiole is restored, airway protection may still be guaranteed [29]. However, to ensure maintenance of sphincteric mechanisms, it is of crucial importance to preserve at least the postero-lateral part of the arytenoid to allow the anatomical integrity of the hypopharyngo-laryngeal wall, thereby minimizing the risk of swallowing disorders [36, 37].

Concerning voice quality, we found no significant post-operative difference in VHI-30 results (*p* = 0.65) and the V item (*p* = 0.31) of the ADVS questionnaire compared to pre-operative measures. This is in accordance with data from the literature [38, 39] and, as shown by Hans et al., objective voice parameters in patients submitted to transoral PCs, despite early worsening, improved after 6 months, which is attributable to compensation mechanisms such as increased supraglottic sphincter closure [40].

All patients in the present cohort reported subjective improvement in respiration under exertion as shown by the D score variation (D_POST_ − D_PRE_) of − 1.45 (CI_95%_ − 1.84, − 1.07; *p* < 0.0001), and all tracheostomized patients were successfully decannulated after a maximum of 18 months. Five out of nine tracheostomized patients were successfully decannulated within 6 months. The remaining four patients had a decannulation delay due to associated comorbidities, in line with what reported in the literature [41].

The postoperative mean PEF% value of 53.7%, with a median of 52.5% (min 12.2%, max 96.0%), clarify that airway patency is far from standardized normal values (≥ 80%) and comparable to patients affected by mild subglottic stenosis [23]. On the other hand, the residual presence of a glottic resistance can explain the still satisfactory subjective phonatory results, underlining that the goal to be achieved for CO_2_ laser PC ± MPA is the best balance between respiratory and phonatory functions.

Furthermore, the possibility of a second intervention must be always taken into account. In our cohort, the risk of re-intervention was up to 15% within the first year after surgery, which appeared to be stable even after 5 years. Among the subgroup of patients who underwent a PC without MPA, the chance to need a second surgery was 27% within the first year. In contrast, adding MPA leads to a trend in reduction in such risk of 9.1%. Every patient who underwent PC without MPA, that requested a revision surgery, was treated within 6 months from the first surgery. On the contrary, patients receiving PC with MPA, who required revision surgery, were treated at least 6 months after the first surgical procedure. A possible explanation of this is ascribable, in the former, to the occurrence of edema of the vocal cord and presence of fresh granulation tissue that can cause a critical respiratory space reduction requiring an early reintervention. On the contrary, in the latter subgroup (PC with MPA), revision surgery was needed to improve the airway patency on a stabilized scar tissue and in one case a contralateral PC was performed. Nonetheless, our bivariate analysis showed that adding MPA does not significantly impact postoperative quality of life, and more specifically does not compromise swallowing function measured by the EAT-10 questionnaire.

## Conclusions

From our analysis, transoral CO_2_ laser posterior cordotomy combined with medial partial arytenoidectomy is a safe, customizable and minimally invasive treatment that can guarantee a good balance between quality of voice and acceptable airway patency without the need of tracheotomy for patients suffering from BAVCP.

## Data Availability

Full dataset will be available upon reasonable request to the corresponding author.
